# Impact of the COVID-19 Pandemic and Psychosocial Coping Strategies in Health Sciences Students at the University of Seville: A Pilot Study

**DOI:** 10.3390/healthcare9121661

**Published:** 2021-11-30

**Authors:** Rocío de-Diego-Cordero, Cristina Martínez-del-Carmen, Patricia Bonilla Sierra, Ana-Magdalena Vargas-Martínez

**Affiliations:** 1 Research Group PAIDI-CTS 969 Innovation in Health Care and Social Determinants of Health, School of Nursing, Physiotherapy and Podiatry, University of Seville, 41009 Seville, Spain; rdediego2@us.es; 2 School of Nursing, Physiotherapy and Podiatry, University of Seville, 41009 Seville, Spain; marmardel11@alum.us.es; 3 Health Sciences School, Private Technical University of Loja, Loja 11-01-608, Ecuador; pbonilla65@utpl.edu.ec

**Keywords:** coronavirus, students, mental health, depression, young people, psychological support

## Abstract

The new infection by coronavirus has supposed a challenge to all health systems worldwide, affecting our psychosocial health. Education as we knew it has changed, which is why university students, attending Health Sciences courses in this case, have been affected by the pandemic. This study aimed to analyze the impact of the preventative measures and restrictions associated with COVID-19 on multiple mental health and psychological well-being indicators in Health Sciences students at the University of Seville. A descriptive and cross-sectional pilot study in the University of Seville by means of an online questionnaire elaborated was conducted. Of the final sample (*n* = 68), more than 60% of the students acknowledged having received specific training by their university and/or health institution where they perform practices on COVID-19 measures; however, they negatively emphasized not having received psychosocial aid or support in most of the cases (94.12%). As the health situation imposed by COVID-19 is considered long-lasting, the proposal is to plan short- and long-term strategies for promotion and intervention in the mental health of students and future health care workers.

## 1. Introduction

Declaration of the pandemic by the World Health Organization (WHO) on 11 March 2020 [1], caused by the new coronavirus, put the entire world out of control. Both science and the health systems burst, the world economy collapsed, and the enormous vulnerability and fragility of mankind was revealed, in particular of those who act in the front line against this virus, including health care professionals and students attending courses in these sciences.

In Spain, the state of alarm was declared on 14 March 2020, through Royal Decree No. 463/2020 [2], which included severe confinement, quarantine, and social isolation measures, with the implementation of actions to limit the virus’ spread given the high morbidity and mortality it was causing at the time. Among these restrictive measures, limitation of people’s circulation freedom was established, including a prolonged confinement situation that severely altered the lifestyle of a large part of the Spanish population. In case of the education sector, the closure of universities and institutions of higher education to contain the spread of the virus prevented the maintenance of university activities in their face-to-face modality and they were specifically online [3]; however, in case of Health Sciences students of University of Seville, they had the possibility to continue their traineeship in the health institutions when the confinement ended.

Some articles have been published about the impact of epidemic crises on health professionals’ mental health [4] and about possible interventions to prevent potential complications [5]. However, little is known about the impact and psychosocial coping strategies of Health Sciences students, who are exposed not only to the pandemic situation but also to work-related stress, high mental burden, professional strain, moral distress, and compassion fatigue or secondary traumatic stress [6].

Levels, work-related stress, risk of contagion, and lack of suitable means, both material and human, to fight against the virus added to the quarantine situation, which limited university attendance and participation in social activities, might cause important harm to mental health, such as anxiety and depression, [7]. Recent studies have detected the onset and experience of depression and anxiety symptoms [8], as well as of Burnout [9] syndrome, in addition to personal and social impacts [10], which could affect their response capacity during the pandemic [11].

## 2. Materials and Methods

### 2.1. Design

A descriptive, correlational, and cross-sectional pilot study was conducted between 15 March 2021 and 15 May 2021, at the University of Seville (US). The state of emergency in Spain was declared on 14 March 2020 and finished 9 May 2021 [12]. During the study period, time restrictions, limited capacity in public spaces, etc. were employed but not lockdown. The end of the academic year 2019/2020 was carried out in the universities in an online mode, and during the academic year 2020/2021, the University of Seville opted for the blended mode with limited capacity in classes, so that students attended some classes online and others in person. It was carried out by means of an online questionnaire that was available to the students for 60 days from initiation of the data collection period. The approximate time to answer the entire survey was 20 min. It included sociodemographic topics and standardized tests to assess multiple indicators related to mental health, psychological well-being, and coping styles. Participation was strictly anonymous and confidential.

Online filling-in of this questionnaire allowed the students to answer it from their electronic devices. In addition, the questionnaires’ web administration minimized the risk of data loss and allowed for automatic and immediate calculation of scores, with consequent time optimization. Further, it is accepted by the general population and ensures anonymity.

All of the above was performed with prior informed consent according to Organic Law No. 3/2018 for the Protection of Personal Data and Guarantee of the Digital Rights, dated 5 December [13], with prior invitation sent via the University of Seville’s institutional email service. No differentiation was made between COVID-19-positive or -negative students and there were no exclusions based on sociodemographic variables, such as gender or ethnicity.

Anonymity was ensured in two ways: by randomizing or omitting the IP information of the device in which the survey was answered and by not asking questions that allowed identification of the participant.

### 2.2. Sample Size, Population, and Study Variables

The sample was collected among undergraduate students attending Health Sciences specialties, such as Nursing, Physiotherapy, and Podiatry, at the University of Seville, and were performing practices in the 2020–2021 academic year, given during the 2019–2020 academic year, when the state of emergency was declared and confinement measures were applied (from March to June 2020), during which the students did not carry out their clinical practices. Collaboration was asked from some professors who taught in such courses to conduct the online survey; they allowed answering of the questionnaire in some of their classes as long as the participants met the criteria.

Since it was a pilot study, the sample size was calculated according to Viechtbauer et al. [14] through the formula proposed by this author to calculate the sample size required to detect a problem in a pilot study. In order to attain a sample that was statistically representative of our target population, according to the latest publication of the US statistical annual report, from a total of 1586 students (930 from Nursing, 384 from Physiotherapy, and 373 from Podiatry), considering a 95% confidence level and 5% of statistical power of the anxiety and/or depression symptoms probability, the total sample required was 59 students. This probability taken into account is lower than that found in other studies to reach a larger sample size and greater precision, given the heterogeneity of our population in relation their practices and their experience during the COVID-19 pandemic.

All students who belonged to the same class group were invited to fill in the questionnaire. A total of 164 questionnaires were initially collected, from which only 68 were valid due to incompleteness.

Of the final sample, with a mean age of 22.76 ± 4.93 years old, more than 80% were women (n = 57). The mean number of household members was around 3 individuals, with no statistically significant differences between men and women. Only 14.71% of the sample lived with an older adult requiring care. It is noted that spirituality was found in 81.8% of the men and 64.9% of the women, although this difference was not statistically significant. The predominant religious belief was the Catholic religion, accounting for 66.18% of the total sample. Regarding perception of health status, 47.06% mentioned good physical health status; however, in the case of mental health, there was a higher percentage among those who indicated that it was fair (38.24%) against 33.83% who reported good mental health status. In relation to the practices and studies of the sample analyzed, 92.65% consisted of Nursing students, who mostly performed their practices in institutions belonging to the public health system with a part-time regime, and who stated having “some” freedom and supervision while performing their practices.

### 2.3. Measuring Instruments

The questionnaire used was divided into 3 major blocks: the first, consisting of sociodemographic questions and related to health sciences students’ practices characteristics; the second, elaborated with questions related to COVID-19 at the personal and family levels and concerning the perceived help from the university; and the third, in which 9 validated scales were used to assess multiple mental health and psychological well-being indicators and coping styles (Table 1).

Regarding health sciences students’ practice characteristics, freedom perceived while performing their practice is understood as the freedom to choose the tasks or procedures to be carried out, schedules, number of clinical cases to attend, etc., and supervision perceived while performing their practice refers to the review by an expert healthcare professional of the tasks or procedures that students perform, the evaluation of their progress, and the support or guidance received.

### 2.4. Data Analysis

A descriptive analysis about the sociodemographic characteristics related to the practices in health institutions and with the experience during the COVID-19 pandemic was performed with the use of mean values and standard deviations in the case of the numerical variables, and resorting to absolute and percentage (%) frequencies for the categorical variables. No analyses by group (sex or another) were performed due to the sample size and difference in proportion between groups. A correlation analysis was performed (Pearson’s when distribution of the variables followed normality, and Spearman’s when that was not the case). The associations between different sociodemographic variables and those related to experience during the COVID-19 pandemic with the variables studied by means of the different scales applied, namely: perceived stress level, risk alcohol consumption, level of psychological distress, psychological resilience in the face of the experiences, self-compassion, spiritual well-being, resilience, and feeling of loneliness, were studied by means of correlation analysis (Pearson’s when the distribution of the variables followed normality, and Spearman’s when that was not the case) since, to such an end, the overall score of each scale was used. These coefficients were classified as: “high degree”: if the coefficient value lies between ± 0.50 and ± 1, then it is said to be a strong correlation; “moderate degree”: if the value lies between ± 0.30 and ± 0.49, then it is said to be a medium correlation; and “low degree”: when the value lies below + 0.29, then it is said to be a small correlation.

Some variables, such as age, were centered by using medians, as no 0 value was included in these variables for the sample under study. The data were analyzed in the R software (version R-3.6.3, GNU General Public License from the Free Software Foundation: https://www.r-project.org/about.html, accessed on 12 October 2021). A *p*-value < 0.05 was accepted as statistically significant.

## 3. Results

Regarding the data related to the COVID-19 pandemic and to the Health Sciences students’ experience, it is noted that more than 60% of them acknowledged having received specific training on this topic by their university and/or health institution where they perform their practices. It is also negatively noted that they did not receive any psychoemotional aid or support in most of the cases (94.12%). In relation to having family members and/or close friends who are at risk of a positive COVID-19 diagnosis or who have died due to diseases related to a positive COVID-19 diagnosis, 21 (30.88%) and 15 (22.06%) students experienced these circumstances, respectively. Despite the pandemic situation, the clinical care provided by most of the students during their internships in health centers was mostly face-to-face (64.71%), and 29.41% of the students that make up the sample reported having taken isolation measures, leaving their family home where they live with their parents during the period of their clinical practice.

Regarding the different scales that were applied to the sample, their mean scores can be seen in Table 2.

Regarding the stress perceived through the PSS-14 tool, the mean score reached a mid-position in relation to the maximum score, since a mean of 28.57 ± 8.84 out of a total of 56 points was obtained.

In relation to the items assessed in the UCLA-R about feelings of loneliness, more than 50% mentioned that they sometimes felt a lack of companionship. Regarding psychological resilience and the risk of suffering anxiety or depression symptoms, through the AAQ-II scale, it is concluded that 40.3% of the students obtained a score equal to or greater than 24, which indicates a predominance of these symptoms and less psychological resilience in the face of life experiences.

This coincides with the PHQ-4 questionnaire, where the same percentage of patients in which there was a predominance of anxiety and/or depression was obtained, with a score equal to or greater than six points in this case. Regarding the resilience level, it is around the scale’s mean score since the maximum possible value is 30.

In relation to risk alcohol consumption, assessed through the AUDIT-C questionnaire, 47.76% were at low risk and 35.82% were at moderate risk.

Regarding satisfaction with life, assessed only through one item from the LSQ scale, it could be asserted that it was high since, out of 7 points, the mean was 6.46 ± 2.34. Finally, regarding self-compassion, it is noted that there were statistically significant differences between men and women, finding more self-compassion among the former.

Regarding the scales, high, positive, and statistically significant correlations weere observed between PSS-14 and AAQ-II, between PSS-14 and PHQ-4, and between PHQ-4 and AAQ-II, as well as between FACIT-SP and SCS. High, although negative, correlations (that is, when the overall score of one scale increases, the other’s decreases) were also observed between PSS-14 and SCS, between PSS-14 and FACIT-SP, between AAQ-II and SCS, and between AAQ-II and FCIT, as well as between PHQ-4 and SCS (Table 3).

Additional correlation analyses were performed to study the association between the different variables assessed by means of the scales and the data collected regarding sociodemographic aspects, experience during the pandemic, and perception of the Health Sciences students about their health status (Table 4 and Table 5).

Regarding the perception of mental health status in the last 30 days, high, negative, and statistically significant correlations were observed between this variable and perceived stress (PSS-14), the risk to suffer anxiety or depression symptoms (AAQ-II and PHQ-4), and so less psychological resilience, indicating that a lower score in these scales is associated with a better perception of mental health status and vice versa, given that a lower score indicates lower perceived stress and a lower risk of suffering anxiety or depression symptoms. High, although positive, correlations were observed between the perception of mental health status and self-compassion and spiritual well-being, indicating that a better perception of mental health is associated with higher levels of compassion and spiritual well-being.

Related to age, only the predominance of anxiety and/or depression symptoms (PHQ-4) and life satisfaction level (LSQ) were found to be associated with this. Given that this correlation was negative, older people scored lower in those scales, indicating a lower predominance of anxiety and/or depression symptoms and lower life satisfaction level.

Regarding spirituality, those who considered themselves spiritual people obtained a lower score in AAQ-II, meaning that they had better psychological resilience in the face of the experiences.

Regarding having family members and/or close friends who are at risk of COVID-19, low-moderate, positive, and statistically significant correlations were observed between this variable and spiritual well-being and alcohol use disorders.

Regarding health sciences students’ practices characteristics, low-moderate, negative, and statistically significant correlations were observed between freedom perceived while performing their practices (understood as the freedom to choose the tasks or procedures to be carried out, schedules, number of clinical cases to attend, etc.) and perceived stress (PSS-14) and the risk of anxiety and/or depression symptoms (AAQ-II and PHQ-4). However, a positive association was observed between freedom perceived while performing their practices and compassion level. These associations were studied given that the stress level of the health sciences student can be increased in a pandemic situation as a consequence of the management in health centers in terms of staffing and lack of knowledge in the management of the disease, resulting in stress of the health professionals who work in the centers [25] who must monitor the progress of the students.

## 4. Discussion

The objective of this study was to analyze the impact of the preventative measures and restrictions associated with the COVID-19 pandemic on multiple mental health and psychological well-being indicators in the Health Sciences course at the University of Seville. The main results indicate that 94.12% acknowledged having received no psychoemotional aid or support in most of the cases from their institution during the COVID-19 pandemic period. Allied to this, a positive correlation is to be noted between stress perception and anxiety and depression, and how all three relate among each other given the scales used. In addition, a positive correlation is evidenced between spirituality and compassion, as well as a negative correlation of stress, anxiety, and depression with spirituality and compassion. Alcohol consumption has increased during the COVID-19 pandemic [26], finding a moderate risk of developing an alcohol dependence in 35.82% of the students. Likewise, an association was evidenced between a bad or very bad perception of health status with more perceived stress, higher distress level, and less psychological resilience and, therefore, greater risk of presenting anxiety and/or depression symptoms. An association was also found between the students with family members and/or close friends at risk of contracting COVID-19 or who died due to the disease and those who were isolated from their parents when compared to the participants who did not implement any isolation measure, with more psychological distress and, therefore, greater predominance of anxiety and/or depression.

Regarding lack of psychoemotional aid or support by their institution during the COVID-19 pandemic period, most of the participants pointed to a lack of support. In relation to that, in early March, a study was conducted considering that young individuals, university students among them, presented the possibility of developing emotional disorders, which is why prevention and intervention programs to reduce stress levels should be devised and promoted by the educational institutions themselves [27]. Allied to the above, another study indicated that early identification of Burnout syndrome symptoms (sleep deprivation, academic overload, imbalance between personal and academic life, life crises during studies, death of a family member, or low perception of self-efficacy, among others) could reduce the number of psychosocial problems [28] that can emerge in academic life, hence the importance of emotional support. At the institutional level, a lack of time for a good diet, rest, and self-care [29] and the use of digital platforms for learning have also been pointed out as factors that generate personal frustration [30].

Regarding stress, previous studies of population quarantine situations showed that there was a psychological impact of relevant intensity, even with long-term effects. During the 2003 SARS epidemic, it was shown that sleep was one of the first aspects affected by stress [31]. However, in addition to the assessed symptoms, sleep changes can constitute a disease by themselves, and it is well-known that they are not only associated with psychiatric disorders but also with alterations in various systems. Recent studies analyzed the impact of the COVID-19 pandemic on the mental health of the population [32], but the information about sleep changes is quite limited.

There are studies that support the results obtained in this intervention that higher compassion and spirituality levels are negatively correlated with the perception of stress, anxiety, and depression. Similarly to the current study, a study conducted among adolescents showed a strong and negative relationship between self-compassion and depression [33]. Other studies also identify men as with a higher level of self-compassion [34] when compared to women [35]. Other previous studies have indicated that compassion for others, by others, and self-compassion are positively correlated with commitment to life, with the possibility of asserting that the greater this commitment, the greater the satisfaction and the lower the anxiety and stress levels [36]. The inclusion of spirituality in assistance provided is essential for comprehensive care [37].

In relation to coping strategies, in a study conducted in Colombia [38], several strategies have been proposed, with maintenance of good eating and sleeping habits among the most noticeable, as supported by other authors. Recent evidence supports that lower adherence to many healthy lifestyles is associated with worse results in several psychiatric disorders [39]. As strategies for the management of emotions, the performance of breathing and mindfulness exercises before initiating and at the end of care activities has been proposed. The UNESCO recommends yoga and meditation as tools to control breathing and help young individuals to improve their anxiety levels. Additionally, in the case of yoga, the opportunity of doing physical exercise contributes even more benefits to the mind [40].

Furthermore, in the specific case of mindfulness practice, it has been proven to be an efficient tool to promote resilience among physicians [41].

Regarding the association between anxiety and/or depression symptoms, isolation, psychological distress, and predominance of anxiety and/or depression, a previous study conducted with Spanish students showed that 62% of the interviewees suffered high anxiety levels [42], a percentage that is similar to the values detected in studies conducted in the general Chinese population during COVID-19 [43]. Regarding the above, previous studies have associated social isolation or confinement with an increase in the feeling of loneliness and with the perception that COVID-19 exerted a greater impact on everyday life, although there is an association between a certain reduction in the feeling of loneliness and greater social support [44]. In the case of Spanish nursing students, a previous study aimed to describe the perceptions and experiences of nursing students who cared for patients with COVID-19 symptoms in the early stages of the outbreak. Their findings showed that many participants experienced feelings of stress, fear, and sadness due to emotional exhaustion caused by the pressure of having to provide adequate care and the lack of experience in certain procedures that had to be performed, which also affected the fear of being infected by a lack of protection, and also infecting family members and feeling lonely when they worked without the supervision of qualified personnel [3].

The findings of the study conducted in the Private Technical University of Loja (Ecuador) are similar: psychological inflexibility and loneliness mediated the impact of stress on anxiety and depression symptoms in participants, regardless of gender and the history of a positive COVID-19 diagnosis [45].

In addition to this, this study showed a positive correlation between the number of hours of practice and less psychological resilience with a certain predominance of presenting anxiety and depression symptoms, which coincides with another study showing that nurses who act on the front line who presented greater resilience with emotional support had fewer anxiety symptoms [46]. Furthermore, other studies identified that the risk of suffering burnout was lower and characterized by a greater use of emotional strategies to reduce stress [25], and another study with physiotherapy study showed a relationship between stress and type D personality and stomatognathic system disorders [47].

Finally, this study had several limitations and strengths that must be acknowledged. In the first place, as the data are cross-sectional, no causality can be established, only associations between variables. Without longitudinal data, it is not possible to show the time precedence of the predictors selected. In the second place, regarding the sample, it is worth noting the sample loss and lack of participation in this type of study, as already pointed out in previous surveys [48]. In addition, the recruitment system and data collection through an online survey limited generalization and hindered real control of the participants. In the third place, as this study was conducted in only one university, it is difficult to generalize its results to other universities or academic curricula.

However, the use of validated questionnaires in Spanish allowed a reduction of some of the possible biases. Allied with this, this study was conducted simultaneously in the Private Technical University of Loja (Ecuador), which allows for a comparative study of both samples. In addition, as this study will be implemented in other universities, it will include data on instrumental social support for students, especially those with symptoms of anxiety and depression.

## 5. Conclusions

Stress and anxiety levels have been high during the last year (from March 2020), for different reasons, with the following standing out: lack of knowledge about the pandemic, fear that a family member could contract COVID-19 and die, prolonged confinement periods throughout the year (from March 2020 to June 2020), and limited emotional support received from the institution. There has also been an increase in the feeling of loneliness, which was reduced with greater social support and in the cases of students who were not isolated from their parents.

As the most noticeable strategy, we identified mindfulness, which can be applied to reduce stress and anxiety levels and improve psychological resilience.

Spirituality and compassion help reduce anxiety and perceived stress levels while also increasing satisfaction with life and enabling greater commitment to it.

As the health situation imposed by COVID-19 is considered long-lasting, the proposal is to plan short- and long-term strategies for promotion and intervention regarding the mental health of students and future health care workers. Therefore, subsequent studies need to devise more specific strategies for emotional support in students.

The results of this study do not provide definitive evidence, because it was a pilot study with a small sample, and although a big effort was made to collect participants (as it explained above), a designed cross-sectional descriptive is needed. However, this study can be considered as a first step in an approach to the impact of the confinement measures associated with COVID-19 on multiple mental health and psychological well-being indicators in Health Sciences students.

## Figures and Tables

**Table 1:** Scales used.

Scales Used and Psychometric Characteristics	Definition	Score—Measurement
**Perceived Stress Scale** **(PSS-14)** [15]. α = 0.81	It assesses the level of perceived stress in the last month	It consists of 14 items with an answer format in a five-point scale (0 = never, 1 = almost never, 2 = occasionally, 3 = often, and 4 = very often). The total score of the PSS scale is obtained by reversing the scores of items 4, 5, 6, 7, 9, 10 and 13 (in the following order: 0 = 4, 1 = 3, 2 = 2, 3 = 1 and 4 = 0) to then add up the 14 items. The direct score obtained indicates that higher values correspond to higher levels of perceived stress.
**UCLA Loneliness Scale (Revised Version, Short Version) UCLA-R** [16] Kaiser-Meyer-Okin (KMO) = 0.839	It assesses the subjective feelings of loneliness, as well as those of social isolation.	It has 20 items, although only 3 have been selected. The participants had to choose one of 4 possible answers: never, rarely, sometimes or always.
**Avoidance and Action Questionnaire II** (AAQ-II) [17]. Kaiser-Meyer-Olkin (KMO) = 0.087	It assesses experiential avoidance or, on the contrary, psychological resilience, depending on the orientation of its ten reagents.	It consists of 7 reagents, each one with seven answer options varying from “never” to “always”, according to the beliefs. Higher total scores mean less resilience, whereas lower overall scores indicate more resilience regarding the experiences went through by each individual.
**Brief Resilience Scale (BRS)** [18] Kaiser-Meyer-Olkin (KMO) = 0.793	It assesses people’s ability to recover from stressful circumstances. This resilience can be particularly important for people who are dealing with stressful life events, such as health-related problems.	It consists of 6 items with a 5-point answer scale varying from 1 (totally disagree) to 5 (totally agree). Higher scores indicate more resilience.
**The Alcohol Use Disorders Identification Test: Self-Report Version (AUDIT-C)** [19,20,21] α = 0.75	It measures excessive alcohol consumption. It can assist in the identification of excessive alcohol consumption as the cause of the current disease. It also provides a framework for the intervention to assist drinkers with harmful or risk consumption in reducing or ceasing alcohol consumption, so that they can avoid the detrimental consequences of consumption.	It has 10 items, each one scored from 0 to 4 points. We have only used three of them. It is scored from 0 to 12, as follows: low risk if below 2, moderate risk between 3 and 5, high risk between 6 and 7, and severe risk between 8 and 12 (for women), only differing in low risk (0–3) and medium risk (4–5) for men.
**Patient Health Questionnaire (PHQ-4)** [22] α = 0.903	It assesses the patients’ level of psychological distress and characterizes their symptoms as of anxiety and/or depression, predominantly.	It consists of 4 items, two of them for screening depression and the other two for screening anxiety. These items have been extracted from two different scales and validated at the international level. Significantly high scores are generally considered as those equal to or greater than half of the maximum score, that is, PHQ-4 ≥ 6.
**Life Satisfaction Questionnaire (LSQ)** [22] Kaiser-Meyer-Olkin (KMO) = 0.847	It assesses perception of quality of life in women with breast cancer. This instrument can also be used in complementary treatments.	It consists of 34 items (in this study, only one of them was selected, whose question is as follows: *Which is your overall level of satisfaction with life?*). It is scored with a value between 1 and 7, where the higher the score, the greater the satisfaction level.
**SCS Compassion Scale in Spanish (12-item version)** [23] Kaiser-Meyer-Olkin (KMO) = 0.79	It assesses an individual’s level of compassion.	It has 26 items. In 2011, a shorter version of the SCS scale was developed, with only 12 items. Coding key: - Self-kindness, items: 5, 12, 19, 23, 26. - Common humanity, items: 3, 7, 10, 15 - Mindfulness, items: 9, 14, 17, 22 - Self-judgment, items: 1, 8, 11, 16, 21 - Isolation, items: 4, 13, 18, 25 - Overidentification, items: 2, 6, 20, 24
**Functional Assessment of Chronic Illness Therapy—Spiritual Well-Being (FACIT-SP)** [24] α = 0.835	It assesses aspects related to spiritual well-being related to the meaning and purpose of life, peace and the sensation of strength and comfort derived from faith and from spiritual beliefs.	It has 12 items. It is evaluated with a 5-point scale, varying from 0 (not at all) to 4 (very much). Higher scores indicate higher levels of spiritual well-being.

**Table 2:** Scores in the scales.

Variables	Total Sample *n* = 68
**Perceived Stress Scale (PSS-14)**	28.57 (8.84)
**UCLA Loneliness Scale (Revised Version, Short Version) UCLA-R**	
*How often do you feel that you lack companionship?*	
Never	7 (10.45)
Rarely	16 (23.88)
Sometimes	35 (52.24)
Always	9 (13.43)
*How often do you feel left out?*	
Never	19 (28.36)
Rarely	26 (38.81)
Sometimes	18 (26.87)
Always	4 (5.97)
*How often do you feel left isolated from others?*	
Never	25 (37.31)
Rarely	26 (38.81)
Sometimes	13 (19.40)
Always	3 (4.48)
**Avoidance and Action Questionnaire II (AAQ-II)**	22.52 (10.37)
≥24 points	27 (40.3)
**Brief Resilience Scale (BRS)**	17.84 (2.47)
**AUDIT-C**	
Low risk (0–2 points for women; 0–3 for men)	32 (47.76)
Moderate risk (3–5 points for women; 4–5 points for men)	24 (35.82)
High risk (6–7 points)	10 (14.93)
Severe risk (8–12 points)	1 (1.49)
**Patient Health Questionnaire (PHQ-4)**	4.99 (3.43)
≥6 points (predominance of anxiety and/or depression)	27 (40.3)
**Life Satisfaction Questionnaire (LSQ)**	
Which is your overall level of satisfaction with life?	6.46 (2.34)
**SCS Compassion Scale in Spanish (12-item version)**	38.15 (9.16)
*Self-kindness subscale*	5.94 (2.13)
*Self-judgment subscale*	6.57 (2.14)
*Common humanity subscale*	5.81 (1.72)
*Isolation subscale*	7.15 (2.52)
*Mindfulness subscale*	6.39 (2.03)
*Overidentification subscale*	6.30 (2.36)
**Functional Assessment of Chronic Illness Therapy—Spiritual Well-Being (FACIT-SP)**	27.05 (6.58)

Note: Mean values and standard deviations are presented in between round brackets for quantitative or numerical variables, and absolute and percentage frequencies are indicated in between round brackets for categorical variables.

**Table 3:** Correlations between the scales.

Scales	PSS-14	AAQ-II	BRS	PHQ-4	LSQ	SCS	FACIT-SP
**PSS-14**	1 (0)						
**AAQ-II**	0.698 ***	1 (0)					
**BRS**	0.101 (0)	0.103 (0)	1 (0)				
**PHQ-4**	0.796 ***	0.772 ***	0.131 (0)	1 (0)			
**LSQ**	−0.251 **	−0.286 **	−0.036 (0)	−0.213 *	1 (0)		
**SCS**	−0.707 ***	−0.697 ***	−0.078 (0)	−0.656 ***	0.157 (0)	1 (0)	
**FACIT-SP**	−0.597 ***	−0.556 ***	−0.044 (0)	−0.485 ***	0.304 **	0.595 ***	1 (0)

***, ** and * represent 1%, 5% and 10% statistical significance. PSS-14: Perceived Stress Scale; AAQ-II: Avoidance and Action Questionnaire II; BRS: Brief Resilience Scale; PHQ-4: Patient Health Questionnaire; LSQ: Life Satisfaction Questionnaire; SCS: Compassion Scale in Spanish; FACIT-SP: Functional Assessment of Chronic Illness Thera-py—Spiritual Well-Being.

**Table 4:** Associations between different psychological outcomes and experience during the COVID-19 pandemic among health sciences students.

	Age	Gender	Spirituality	Perception of Physical Health Status (Last 30 Days)	Perception of Mental Health Status (Last 30 Days)	Family Members and/or Close Friends Who Are at Risk of COVID-19	Family Members and/or Close Friends Who Have Died due to COVID-19	Changes IMPLEMENTED since the Beginning of the Pandemic
**PSS-14**	−0.170 (−0.398; 0.077)	0.113 (−0.134; 0.348)	−0.139 (−0.370; 0.109)	−0.204 (−0.426; 0.042)	−0.669 (−0.785; −0.508) ***	−0.180 (−0.406; 0.066)	−0.011 (−0.254; 0.234)	0.074 (−0.173; 0.313)
**AAQ-II**	−0.155 (−0.381; 0.088)	−0.036 (−0.274; 0.206)	−0.241 (−0.455; −0.001) **	−0.176 (−0.399; 0.067)	−0.728 (−0.824; −0.592) ***	−0.160 (−0.385; 0.084)	−0.052 (−0.288; 0.191)	0.102 (−0.142; 0.334)
**PHQ-4**	−0.250 (−0.462; −0.010) **	−0.037 (−0.275; 0.205)	−0.145 (−0.372; 0.099)	−0.122 (−0.352; 0.122)	−0.744 (−0.835; −0.613) ***	−0.189 (−0.411; 0.054)	−0.040 (−0.277; 0.202)	0.134 (−0.110; 0.363)
**SCS**	0.124 (−0.119; 0.354)	−0.236 (−0.451; 0.0334) *	0.262 (0.024; 0.473) *	0.112 (−0.132; 0.343)	0.650 (0.485; 0.770) ***	0.222 (−0.019; 0.439) *	0.003 (−0.237; 0.243)	−0.142 (−0.370; 0.101)
**BRS**	−0.213 (−0.431; 0.029) *	−0.177 (−0.401; 0.066)	0.112 (−0.132; 0.343)	0.153 (−0.091; 0.379)	0.060 (−0.183; 0.296)	0.017 (−0.224; 0.256)	0.109 (−0.135; 0.340)	−0.065 (−0.300; 0.178)
**LSQ**	−0.261 (−0.472; −0.022) **	0.088 (−0.155; 0.322)	0.148 (−0.095; 0.375)	0.085 (−0.158; 0.319)	0.226 (−0.015; 0.442) *	0.067 (−0.176; 0.302)	−0.107 (−0.338; 0.137)	−0.191 (−0.413; 0.051)
**FACIT-SP**	0.116 (−0.136; 0.353)	−0.150 (−0.384; 0.101)	**NA**	0.169 (−0.083; 0.400)	0.513 (0.303; 0.675) ***	0.361 (0.124; 0.559) **	0.160 (−0.091; 0.392)	0.046 (−0.204; 0.291)
**AUDIT**	−0.074 (−0.309; 0.169)	−0.142 (−0.370; 0.102)	0.111 (−0.133; 0.342)	0.067 (−0.176; 0.302)	0.143 (−0.100; 0.371)	0.253 (0.013; 0.465) **	−0.001 (−0.242; 0.239)	0.015 (−0.226; 0.255)

***, ** and * represent 1%, 5% and 10% statistical significance. NA: Not Applicable. *Note:* Coefficient values and 95%CI are presented in between round brackets. The categorical variables have been recoded to a numerical variable to carry out the correlation analyses: Gender: (1: woman; 0: man). Spirituality: (1: Yes; 0: No). Perception of physical health status (last 30 days): (2: “Good or very good”; 1: “Fair”; 0: “Bad or very bad”). Perception of mental health status (last 30 days): (2: “Good or very good”; 1: “Fair”; 0: “Bad or very bad”). Family members and/or close friends who are at risk of COVID-19: (1: Yes; 0: No). Family members and/or close friends who have died due to COVID-19: (1: Yes; 0: No). Changes implemented since the beginning of the pandemic: (2: “I moved”; 1: “Isolation from my parents”; 0: “None”).

**Table 5:** Associations between different psychological outcomes and Health Sciences students’ practices.

	Number of Weekly Hours in the Health Institution	Freedom Perceived while Performing Your Practices	Supervision Perceived while Performing Your Practices
**PSS-14**	−0.104 (−0.340; 0.143)	−0.408 (−0.593; −0.183) ***	−0.101 (−0.337; 0.146)
**AAQ-II**	−0.039 (−0.276; 0.203)	−0.323 (−0.522; −0.089) **	0.067 (−0.176; 0.303)
**PHQ-4**	−0.006 (−0.246; 0234)	−0.270 (−0.479; −0.032) **	0.054 (−0.189; 0.291)
**SCS**	0.099 (−0.145; 0.331)	0.286 (0.049; 0.492) **	−0.085 (−0.319; 0.159)
**BRS**	−0.231 (−0.446; 0.010) *	−0.169 (−0.394; 0.074)	−0.204 (−0.423; 0.038) *
**LSQ**	−0.027 (−0.266; 0.215)	0.051 (−0.192; 0.287)	0.046 (−0.197; 0.282)
**FACIT-SP**	0.038 (−0.211; 0.283)	0.110 (−0.141; 0.349)	−0.178 (−0.408; 0.073)
**AUDIT**	−0.054 (−0.291; 0.188)	−0.167 (−0.392; 0.076)	0.064 (−0.179; 0.300)

***, ** and * represent 1%, 5% and 10% statistical significance. *Note:* Coefficient values and 95%CI are presented in between round brackets. The categorical variables have been recoded to numerical variable to carry out the correlation analyses: Number of weekly hours in the health institution: 0: Between 0 and 9 weekly hours or Other; 1: Part time (between 10 and 34 weekly hours); 2: Full time (at least 35 weekly hours). Freedom perceived while performing your practices: 3: A lot; 2: Some; 1: Little or Very little; 0: None. Supervision perceived while performing your practices: 3: A lot; 2: Some; 1: Little or Very little; 0: None.

## Data Availability

The authors declare to be responsible for conducting the protocols established by their respective centers to evaluate the volunteers included in the study for scientific research and disclosure purposes and ensure that the requirement of having informed all the study subjects has been complied with, that the participants’ informed consent to participate in the study has been obtained in writing, and that they are in possession of such documents. Therefore, before their incorporation to this study, all the participants were verbally informed about the procedure that was conducted and they digitally agreed with the informed consent to participate in the research and answer the survey. The main researcher explained the study purpose to them, they understand that their participation is voluntary and that they can exit the questionnaire at their will without having to give any explanations. The authors declare that, in this research, the participants’ data privacy was ensured and state that the published paper does not infringe the normative regarding personal data protection, safeguarding the subjects’ identity, since the IP addresses were randomized. The participants consented to the computerized treatment of the data obtained for scientific purposes, according to the legal rules, as per Organic Law No. 3/2018 regarding Protection of Personal Data and Guarantee of the Digital Rights.
